# *In vivo* Mononuclear Phagocyte Migration: Paradoxical Effect of Adrenalectomy

**DOI:** 10.1155/S0962935194000372

**Published:** 1994

**Authors:** N. E. Heluy-Neto, F. Q. Cunha, S. H. Ferreira

**Affiliations:** Department of Pharmacology Faculty of Medicine (Ribeirão Preto) USP Av. Bandeirantes 3900 Ribeirão Preto SP 14049-000 Brazil

## Abstract

The effect of adrenalectomy on neutrophil and monocyte migration into rat peritoneal and pleural cavities was investigated. Carrageenin- or thioglycollate-induced neutrophil emigration into both cavities was enhanced by adrenalectomy. In contrast, monocyte migration into peritoneal cavities induced by these two stimuli was significantl decreased. In pleural cavities, adrenalectomy enhanced the monocyte migration induced by carrageenin but had no effect on that induced by thioglycollate. Administration of physiological doses of glucocorticoids reversed the effect of adrenalectomy on monocyte migration by both stimuli into both cavities. The results support the hypothesis that endogenous glucocorticotds negatively control neutrophil migration independently of the site or type of stimulus. Their role in monocyte migration is, however, dependent on the site of injury and on the type of inflammatory stimulus. There is no obvious explanation for the divergent influence of endogenous glucocorticoids on the monocyte emigration into peritoneal and pleural cavities observed with different stimuli.


Mediators of Inflammation 3, 275–279 (1994)

### *In vivo* mononuclear phagocyte migration: paradoxical effect of adrenalectomy

N. E. Heluy-Neto, F. Q. Cunha and S. H. Ferreira[CA]

Department of Pharmacology, Faculty of Medicine (Ribeirão Preto) USP, Av. Bandeirantes 3900, 14049-000, Ribeirão Preto, SP, Brazil

[CA]Corresponding Author

**The effect of adrenalectomy on neutrophil and monocyte migration into rat peritoneal and pleural cavities was investigated. Carrageenin- or thioglycollate-induced neutrophil emigration into both cavities was enhanced by adrenalectomy. In contrast, monocyte migration into peritoneal cavities induced by these two stimuli was significantly decreased. In pleural cavities, adrenalectomy enhanced the monocyte migration induced by carrageenin but had no effect on that induced by thioglycollate. Administration of physiological doses of glucocorticoids reversed the effect of adrenalectomy on monocyte migration by both stimuli into both cavities. The results support the hypothesis that endogenous glucocorticoids negatively control neutrophil migration independently of the site or type of stimulus. Their role in monocyte migration is, however, dependent on the site of injury and on the type of inflammatory stimulus. There is no obvious explanation for the divergent influence of endogenous glucocorticoids on the monocyte emigration into peritoneal and pleural cavities observed with different stimuli.**

**Key words:** Adrenalectomy, Endogenous glucocorticoids, Inflammation, Monocyte migration, Neutrophil migration

### Introduction

It is generally assumed that migration of phagocytes (neutrophils or monocytes) into the various body cavities or tissues is governed by factors released by inflammatory stimuli. This assumption is supported by a great number of studies on neutrophil migration,[1-3] including those which show that during the initial stages of the inflammatory process an increase in glucocorticoid (GCC) secretion by the adrenal glands inhibits neutrophil migration, independently of the site of inflammation.[4,5] In acute inflammation, GCC secretion is induced by the hypothalamo-pituitary-adrenal (HPA) axis through the influence of factors released in the inflamed area.[6] Among these factors, cytokines such as interleukin-1 (IL-1),[7,8] IL-6[9] or tumour necrosis factor (TNF)[10] have been shown to be involved. IL-1, in addition to its effect on the HPA axis, also has an effect on the adrenal gland, to directly stimulate GCC secretion.[11] The enhancement of neutrophil migration by inflammatory stimuli in adrenalectomized (ADX)[4] or in animals pretreated with GCC antagonists such as RU 486[5] may be explained by the diminished capacity of the body cells to release lipocortins or by facilitation of the release of chemotactic cytokines. The lipocortins,[12,13] which belong to the superfamily of calcium binding proteins termed annexins,[14] have anti-phospholipase $A_2$ activity.[15,16] GCC induce the release of these proteins and therefore reduce the biosynthesis of leukotrienes thought to be involved in the control of neutrophil migration. In this context, it is interesting to note that in ADX animals there is a reduction in the amounts of both lipocortin-1 and lipocortin-1 mRNA in the body fluids, and that the physiological replacement of corticosterone restores the normal levels of both these components.[17,18] Moreover, in ADX animals, Perreti *et al.* demonstrated that macrophages release more IL-1 and prostaglandin $E_2$ than do macrophages from sham-operated (SHO) ones.[19] Conversely, GCC inhibited the *in vivo* neutrophil recruiting activity on pro-inflammatory cytokines such as interleukin IL-1,[20,21] IL-6,[22] IL-8[23] and TNF.[24,25]

To date, the studies investigating the role of endogenous GCC on leukocyte migration into inflammatory sites have focused on neutrophil migration.[4,5,26] In this paper, we have compared the effect of adrenalectomy on neutrophil and mononuclear phagocyte migration into two different sites of inflammation (the pleural and peritoneal cavities) induced by two different stimuli (carrageenin or thioglycollate). The results confirm the observations of other authors that adrenalectomy potentiates neutrophil migration into peritoneal or pleural cavities. However, with regard to monocyte migration, the response to adrenalectomy depended heavily on

© 1994 Rapid Communications of Oxford Ltd the cavity tested. Adrenalectomy reduced the migration into peritoneal cavities induced by both carrageenin (Cg) and thioglycollate (Tg), but had no effect or enhanced the monocyte migration into pleural cavities induced by Tg and Cg, respectively.

### Materials and Methods

*Drugs and chemicals:* Carrageenin and thioglycollate were purchased from Marine Colloids Inc. and Merck Laboratories, respectively. The steroid hormones oestra-1,3,5(10)-trien-3,17 diol (β-oestradiol) and corticosterone were obtained from Sigma Chemical Company, St Louis, MO. Dexamethasone 21-acetate (Decadronal) was from Merck Sharp & Dohme. All other reagents were purchased from Sigma Chemical Company (USA).

*Animals:* Adult male Wistar rats weighing 150–180 g obtained from the Central Animal House of the Faculty of Medicine (Ribeirão Preto) were used in all experiments. The animals received water and food *ad libitum*.

*Bilateral adrenalectomy:* The animals were surgically prepared 7 days before the experiments. Following light ether anaesthesia, the rats of one group had both of their adrenal glands completely removed (ADX animals), while those of a second group underwent the same process to induce surgical stress, but were left with their glands intact (SHO animals). The ADX rats received an additional supply of saline solution (NaCl, 0.9% w/v) to drink. At the end of the experiments, the ADX animals in which there was macroscopic evidence of remnant adrenal tissue were rejected. The inflammatory stimuli were injected on the seventh day after surgery.

*Experimental models of inflammation:* The neutrophil and mononuclear cell migration into peritoneal and pleural cavities were investigated in SHO and ADX animals. In some experiments, the ADX animals were treated with steroid hormones.

*(a) Peritoneal cavity.* Cg resuspended in 3 ml of phosphate buffered saline (PBS) or 5 ml of thioglycollate (Tg, 3% w/v), was injected intraperitoneally. The migration of neutrophils was analysed 4 h after the injection of Cg (300 μg/rat). While the migration of mononuclear cells was analysed 48, 72 or 96 h after the injection of Cg (500 μg/rat) or Tg (150 mg/rat).

*(b) Pleural cavity.* In pleural cavities, neutrophil migration was also analysed 4 h after the intrapleural injection of Cg (300 μg/ml/rat) and mononuclear cell migration was analysed 48, 72 or 96 h after Cg (500 μg/ml/rat) or Tg (40 mg/ml/rat) injection.

*(c) Collection of exudates.* At selected intervals, the cells which had migrated were harvested from the peritoneal and pleural cavities. For this, the animals were anaesthetized with ether and exsanguinated. Both naïve and stimulated peritoneal and pleural cavities were washed with 10 ml and 5 ml of heparinized PBS (5 U/ml), respectively. In some experiments, the peritoneal cavities were washed with 10 ml of trypsin diluted in PBS (TRY, 1.25% v/v). Total and differential cell counts of the collected washings were estimated as previously described by Souza and Ferreira.[27] The characterization of macrophages (esterase positive cells) performed using a nonspecific esterase staining technique described by Yam *et al.*[28] The results were expressed as the number of mononuclear or esterase positive cells per cavity.

*Leukogram count:* Total leukocyte, neutrophil and mononuclear cell counts were performed on blood samples obtained from the tail vein of ADX or SHO rats which had or had not been treated with Tg (150 mg/5 ml/rat, i.p.) 48 h before. The results were recorded as the number of cells per ml of blood.

*Administration of steroid hormones:* Subcutaneous doses of 2, 20, 200, 2 000 and 6 000 μg/kg of oestradiol (ST) and corticosterone (CT) dissolved in peanut oil, and dexamethasone (DX) dissolved in sterile saline, were injected daily from the sixth day after surgery until the day mononuclear cell migration was measured. The control animals were injected with vehicle solution.

*Statistical analysis:* The results are expressed as the mean standard error of the mean (S.E.M.) and statistical comparisons between the SHO and ADX groups were done using Student's *t*-test. $p$ values $< 0.05$ were considered to be statistically significant.

### Results

Neutrophil migration into peritoneal cavities measured 4 h after Cg injection was significantly enhanced by adrenalectomy (Fig. 1A). In contrast, mononuclear phagocyte migration evaluated 48, 72 and 96 h after Cg injection was significantly reduced in ADX rats compared with SHO animals (Fig. 1B). A similar pattern of mononuclear phagocyte migration was observed when Tg instead of Cg was injected into the abdominal cavities (Fig. 2).

In the pleural cavities, both neutrophil and mononuclear phagocyte migration induced by Cg were enhanced in ADX animals (Fig. 3A and B). However, the mononuclear phagocyte migration into these cavities induced by Tg was not affected by adrenalectomy (Fig. 4). The number of resident mononuclear phagocytes present in the non-stimulated peritoneal or pleural cavities was not modified by adrenalectomy (cross-hatched bars in all figures).

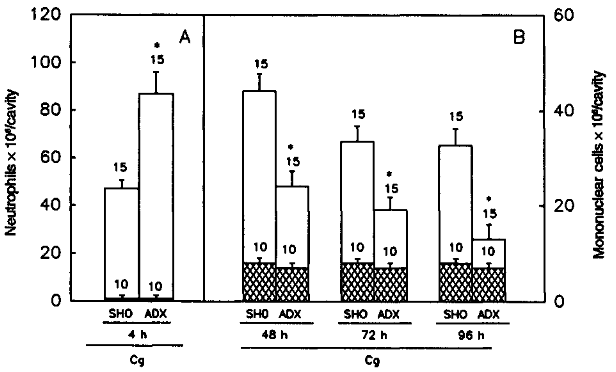

FIG. 1. Adrenalectomy potentiates carrageenin induced neutrophil migration but inhibits mononuclear cell migration into peritoneal cavities. The bars show the number of neutrophils (panel A) and the number of mononuclear cells (panel B) in naïve cavities (cross-hatched bars) and in cavities pre-stimulated (open bars) with Cg (300 μg/cavity, panel A) or (500 μg/cavity, panel B), in sham-operated (SHO) or adrenalectomized (ADX) rats. The values are the means ± S.E.M. of the number of animals indicated above each bar. Asterisks indicate significant differences between the SHO and ADX groups ($p < 0.05$).

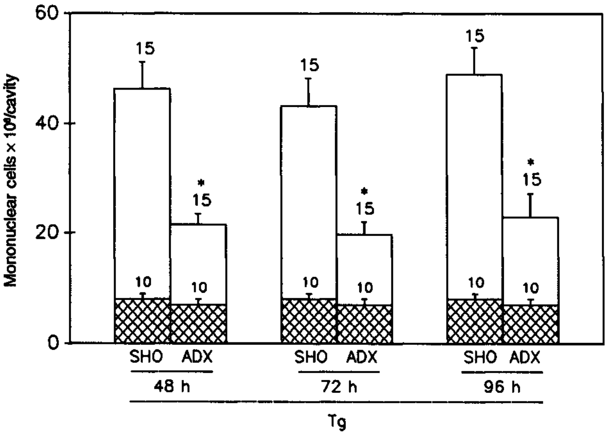

FIG. 2. Adrenalectomy inhibits thioglycollate induced mononuclear cell migration into rat peritoneal cavities. The bars show the number of mononuclear cells collected from naïve cavities (cross-hatched bars) or from cavities pre-stimulated with Tg (150 mg/cavity) 48, 72 and 96 h before (open bars) in sham-operated (SHO) or adrenalectomized (ADX) rats. The values are the means ± S.E.M. of the number of animals indicated above each bar. Asterisks indicate significant differences between the SHO and ADX groups ($p < 0.05$).

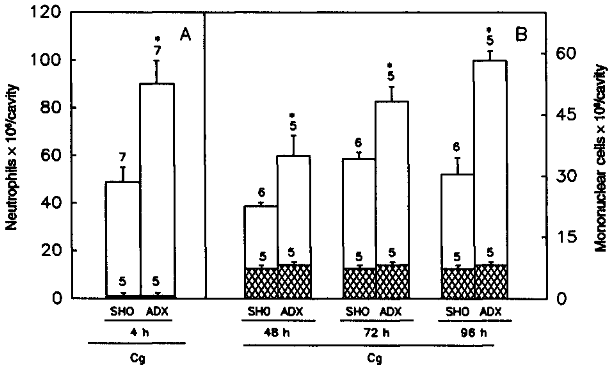

FIG. 3. Adrenalectomy potentiates carrageenin induced neutrophil and mononuclear cell migration into rat pleural cavities. The bars show the number of neutrophils (panel A) and the number of mononuclear cells (panel B) in naïve cavities (cross-hatched bars) and in cavities pre-stimulated (open bars) with Cg (300 μg/cavity, panel A) or (500 μg/cavity, panel B), in sham-operated (SHO) or adrenalectomized (ADX) rats. The values are the means ± S.E.M. of the number of animals indicated above each bar. Asterisks indicate significant differences between the SHO and ADX groups ($p < 0.05$).

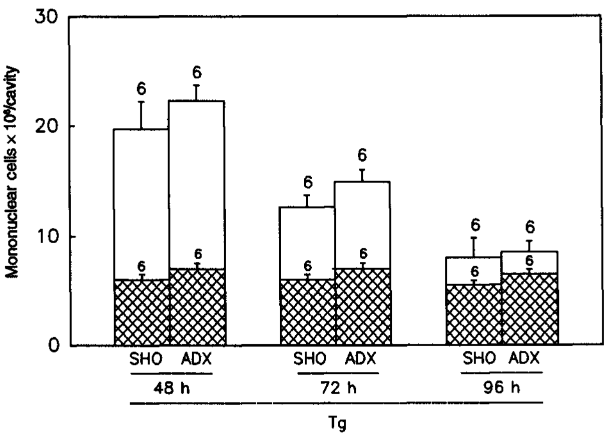

FIG. 4. Adrenalectomy does not alter the thioglycollate induced mononuclear cell migration into rat pleural cavities. The bars show the number of mononuclear cells collected from naïve cavities (cross-hatched bars) and from cavities pre-stimulated with Tg (150 mg/cavity) 48, 72 and 96 h before (open bars) in sham-operated (SHO) and adrenalectomized (ADX) rats. The values are the means ± S.E.M. of the number of animals indicated above each bar. There were no significant differences in the migratory responses of mononuclear cells in the SHO and ADX groups.

Regardless of whether they were prestimulated or not with Tg, all ADX animals had higher leukocyte, neutrophil and mononuclear cell counts than did SHO rats (Table 1).

Daily treatment of ADX animals with DX (2 – 2000 μg/kg) resulted in a bell-shaped curve in which a low dose of DX (20 μg/kg) restored the Tg-induced mononuclear phagocyte migration into peritoneal cavities to levels similar to those observed in SHO animals, while a pharmacological dose (2 000 μg/kg) significantly enhanced the reduction in mononuclear phagocyte migration. Daily treatment with CT also dose dependently reverted the mononuclear cell migration into the peritoneal cavities of ADX animals. At none of the doses studied did ST have any effect on the mononuclear phagocyte migration in ADX rats (Fig. 5).

### Discussion

The results presented above agree with previous experiments showing that adrenalectomy or pretreatment with RU 486 enhances the neutrophil migration induced by inflammatory stimuli such as Cg into pleural cavities and subcutaneous air pouches.[4,5] In addition, a greater inflammatory exudation in ADX animals (data not shown) was observed. These responses are in line with the suggestion that during the acute inflammatory responses the release of GCC by the adrenal cortex partially down-regulates neutrophil migration and exudation via a reduced release of chemotactic cytokines[19] or arachidonic acid metabolites.[4,5]

Paradoxically, adrenalectomy reduced monocyte migration into peritoneal cavities by both stimuli utilized but did not affect or enhance migration into

**Table 1.** Leukogram of sham-operated (SHO) and adrenalectomized (ADX) rats in the presence or absence of thioglycollate (Tg)[a] pre-stimulation

| Cell type | Cell counts[b] | | | |
|---|---|---|---|---|
| | Non-stimulated | | Tg stimulated | |
| | SHO | ADX | SHO | ADX |
| Total leukocytes | 9.7 ± 0.7 | 22.1 ± 2.3* | 7.5 ± 0.9 | 19.1 ± 0.5* |
| Neutrophils | 3.8 ± 1.0 | 7.0 ± 1.1* | 1.9 ± 0.3 | 6.6 ± 1.0* |
| Mononuclear cells | 5.7 ± 2.2 | 14.1 ± 2.2* | 5.0 ± 0.5 | 12.5 ± 0.5* |

[a] Thioglycollate (150 mg/rat) was injected intraperitoneally 48 h before.
[b] Expressed as the number of cells × $10^{-6}$ · $ml^{-1}$. The values are means ± S.E.M. of six observations.
* Significant difference between SHO and ADX groups ($p < 0.05$, Student's *t*-test).

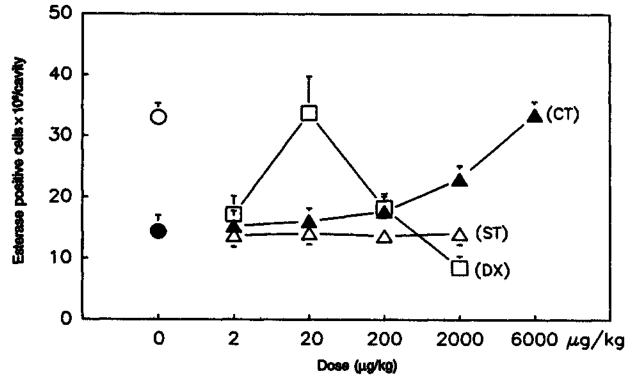

FIG. 5. Dexamethasone (DX) and corticosterone (CT), but not oestradiol (ST), revert the inhibitory effect of adrenalectomy on thioglycollate induced monocyte migration into rat peritoneal cavities. The figure shows the number of esterase positive cells in cavities stimulated with Tg 48 h before in sham-operated (O) and adrenalectomized (■) rats, which received daily subcutaneous doses of DX (□), CT (▲) or ST (△). The values are the means ± S.E.M. of the number of animals indicated above each bar. There were significant differences between SHO and ADX groups ($p < 0.05$, Student's *t*-test), except in the groups of ADX rats which received daily doses of DX (20 µg/kg) and ST (2 000 and 6 000 µg/kg).

the pleural cavities. The reduction of monocyte migration into the peritoneal cavities of ADX animals resulted from the absence of circulating GCC since it was reverted by daily treatment of the ADX animals with low doses of dexamethasone or corticosterone, but not by oestradiol. The reduction of mononuclear phagocyte migration into the peritoneal cavity of ADX animals was not caused by monocytopenia since adrenalectomy produced intense leukocytosis with monocytosis in animals which had or had not been stimulated with Tg. The reduction in the number of migrating monocytes in ADX animals could be due to an increased adherence of these cells to the abdominal wall. However, this possibility can be discarded since the addition of trypsin to the washing fluid did not increase the number of migrating monocytes in ADX animals (data not shown). Pharmacological (200 and 2 000 µg/kg) doses of dexamethasone which are able to inhibit neutrophil migration[29,30] inhibited monocyte migration into the peritoneal cavities of SHO rats (data not shown) and enhanced the reduction in monocyte migration already observed in ADX animals (Fig. 5).

The differential effect of the stimuli in both cavities excludes a possible action of GCC on the monocytes. A simple explanation for the effect of adrenalectomy on monocyte migration into peritoneal cavities is that GCC, by a permissive effect, at physiological concentrations facilitates the release of chemotactic factors for monocytes by resident peritoneal cells. Should this be the case, this permissive effect would be under the influence of the microenvironment, since adrenalectomy had either no effect or enhanced the migration into pleural cavities induced by inflammatory stimuli. We have no explanation for the selective action of Tg or Cg on monocyte migration into the pleural cavities of ADX animals. This enhancement of monocyte migration could be abolished by daily treatment of the ADX animals with small doses of dexamethasone. Similarly, the treatment of SHO or ADX animals with pharmacological doses of dexamethasone also diminished the migration induced by Cg. These results indicate that pharmacological concentrations of GCC inhibit the release of a chemotactic factor for monocytes by pleural or peritoneal cells.

In conclusion, the results confirmed that endogenous GCC play an important role in modulating the acute events of the inflammation, by exerting a negative control on neutrophil migration and exudation which is independent of the site of injury. On the other hand, the effect of endogenously released corticoids on monocyte migration is dependent on the site of injury and on the type of inflammatory stimuli. Furthermore, the results clearly indicate that observations regarding monocyte migration in one tissue or cavity cannot be generalized to other tissues.

### References

1. Wilkinson PC. Cellular accumulation and inflammation. In: Dale MM, Foreman JC, eds. *Textbook of Immunopharmacology* (2nd edition). Oxford: Blackwell Scientific Publications, 1989; 209–222.
2. Faccioli LH, Souza GEP, Cunha FQ, Poole S, Ferreira SH. Recombinant interleukin-1 and tumor necrosis factor induce neutrophil migration *in vivo* by indirect mechanisms. *Agents Actions* 1990; **30**: 344–349.
3. Ribeiro RA, Flores CA, Cunha FQ, Ferreira SH. IL-8 causes *in vivo* neutrophil migration by a cell-dependent mechanism. *Immunology* 1991; **73**: 472–477.
4. Flower RJ, Parente L, Persico P, Salmon JA. A comparison of the acute inflammatory response in adrenalectomised and sham-operated rats. *Br J Pharmacol* 1986; **87**: 57–62.

5. Laue L, Kawai S, Brandon DD, *et al.* Receptor-mediated effects of glucocorticoids on inflammation: enhancement of the inflammatory response with a glucocorticoid antagonist. *J Steroid Biochem* 1988; **29**: 591–598.
6. Garcia-Leme J, Schapoval EES. Stimulation of hypothalamo–pituitary–adrenal axis by compounds formed in inflamed tissue. *Br J Pharmacol* 1975; **53**: 75–83.
7. Besedovsky H, Del Rey A, Sorkin E, Dinarello CA. Immunoregulatory feedback between interluekin-1 and glucocorticoid hormones. *Science* 1986; **233**: 652–654.
8. Sapolsky R, Rivier C, Yamamoto G, Plotsky P, Vale W. Interleukin-1 stimulates secretion of hypothalamic corticotropin-releasing factor. *Science* 1987; **238**: 522–524.
9. Naitoh V, Fukata J, Tominaga T, *et al.* Interleukin-6 stimulates the secretion of adrenocorticotropic hormone in conscious, freely moving rats. *Biochem Biophys Res Commun* 1988; **155**: 1459–1463.
10. Sharp BM, Matta SG, Peterson PK, Newton R, Chag C, MacCallen K. Tumor necrosis factor-α is a potent ACTH secretagogue: comparison to interleukin-1. *Endocrinology* 1989; **124**: 3131–3133.
11. Roh M, Drazenovich KA, Barbose J, Dinarello CA, Cobb CF. Direct stimulation of adrenal cortex by interleukin-1. *Surgery* 1987; **102**: 140–146.
12. Hirata F. The role of lipocortins in cellular function as a second messenger of glucocorticoids. In: Schleimer RP, Claman HN, Oronsky A, eds. *Antiinflammatory Steroid Action—Basic and Clinical Aspects.* San Diego: Academic Press Inc., 1989; 67–95.
13. Peers SH, Flower RJ. The role of lipocortin in corticosteroid actions. *Ann Rev Resp Dis* 1990; **141**: S18–S21.
14. Duval D, Freuss-Beguin M. Glucocorticoids and prostaglandin synthesis: we cannot see the wood for the trees. *Prostagland Leukot, Essent Fatty Acids* 1992; **45**: 85–112.
15. Blackwell GJ, Carnuccio R, Di Rosa M, *et al.* Glucocorticoids induce the formation and release of anti-inflammatory and anti-phospholipase proteins into the peritoneal cavity of the rat. *Br J Pharmacol* 1982; **76**: 185–194.
16. Errasfa M, Russo Marie F. A purified lipocortin shares the anti-inflammatory effect of glucocorticosteroids *in vivo* in mice. *Br J Pharmacol* 1989; **97**: 1051–1058.
17. Vishwanath BS, Frey FJ, Bradbury M, Dillman MF, Frey BM. Adrenalectomy decreases lipocortin-1 messenger ribonucleic acid and tissue protein content in rats. *Endocrinology* 1992; **130**: 585–591.
18. Peers SH, Smillie F, Elderfield AJ, Flower RJ. Glucocorticoid and non-glucocorticoid induction of lipocortins (anexins) 1 and 2 in rat peritoneal leucocytes *in vivo*. *Br J Pharmacol* 1993; **108**: 66–72.
19. Perreti M, Becherucci C, Scapigliati G, Parente L. The effect of adrenalectomy on interleukin-1 release *in vitro* and *in vivo*. *Br J Pharmacol* 1989; **98**: 1137–1142.
20. Snyder DS, Unanue ER. Corticosterone inhibits murine macrophage Ia expression and interleukin-1 production. *J Immunol* 1982; **129**: 1803–1805.
21. Staruch MJ, Wood DD. Reduction of serum interleukin-1-like activity after treatment with dexamethasone. *J Leukocyte Biol* 1985; **37**: 193–207.
22. Waage A, Slupphaug G, Shlaby R. Glucocorticoids inhibit the production of Il-6 from monocytes, endothelial cells and fibroblasts. *Eur J Immunol* 1990; **20**: 2439–2443.
23. Antilla HSI, Reitamo S, Ceska M, Hurme M. Signal transduction pathways leading to the production of IL-8 by human monocytes are differentially regulated by dexamethasone. *Clin Exp Immunol* 1992. **89**: 509–512.
24. Waag A, Bakke O. Glucocorticoids suppress the production of tumor necrosis factor by lipopolysaccaride-stimulated human monocytes. *Immunology* 1988; **63**: 299–302.
25. Barber AE, Coyle SM, Marano MA, *et al.* Glucocorticoid therapy alters hormonal and cytokine responses to endotoxin in man. *J Immunol* 1993; **150**: 1999–2006.
26. Garcia-Leme J, Farsky SP. Hormonal control of inflammatory responses. *Mediat Inflamm* 1993; **2**: 181–198.
27. Souza GEP, Ferreira SH. Blockade by antimacrophage serum of the migration of pmn-neutrophils into inflamed peritoneal cavity. *Agents Actions* 1985; **17**: 97–103.
28. Yam LT, Li CY, Crosby WH. Cytochemical identification of monocytes and granulocytes. *Am J Clin Path* 1971; **55**: 283–290.
29. Kurihara A, Ohuchi K, Tsurufuji S. Reduction by dexamethasone of chemotactic activity in inflammatory exudates. *Eur J Pharmacol* 1984; **101**: 11–16.
30. Tsurufuji S, Kurihara A, Ojima F. Mechanisms of antiinflammatory action of dexamethasone: blockade by hydrocortisone mesylate and actinomycin D of the inhibitory effect of dexamethasone on leukocyte infiltration in inflammatory sites. *J Pharmacol Exp Ther* 1984; **229**: 237–243.

ACKNOWLEDGEMENTS. We thank Mr Rubens de Mello and Ms I. R. Santos for technical assistance. Nicolau E. Heluy-Neto was supported by a fellowship from FAPESP and CAPES. This work was supported by FAPESP (Brazil).

**Received 7 February 1994; accepted 30 March 1994**
